# Effects of Land Use on Lake Nutrients: The Importance of Scale, Hydrologic Connectivity, and Region

**DOI:** 10.1371/journal.pone.0135454

**Published:** 2015-08-12

**Authors:** Patricia A. Soranno, Kendra Spence Cheruvelil, Tyler Wagner, Katherine E. Webster, Mary Tate Bremigan

**Affiliations:** 1 Department of Fisheries and Wildlife, Michigan State University, East Lansing, Michigan, United States of America; 2 Lyman Briggs College, Michigan State University, East Lansing, Michigan, United States of America; 3 U.S. Geological Survey, Pennsylvania Cooperative Fish & Wildlife Research Unit, Pennsylvania State University, University Park, Pennsylvania, United States of America; 4 School of Natural Sciences, Department of Zoology, Trinity College, Dublin, Ireland; University of Siena, ITALY

## Abstract

Catchment land uses, particularly agriculture and urban uses, have long been recognized as major drivers of nutrient concentrations in surface waters. However, few simple models have been developed that relate the amount of catchment land use to downstream freshwater nutrients. Nor are existing models applicable to large numbers of freshwaters across broad spatial extents such as regions or continents. This research aims to increase model performance by exploring three factors that affect the relationship between land use and downstream nutrients in freshwater: the spatial extent for measuring land use, hydrologic connectivity, and the regional differences in both the amount of nutrients and effects of land use on them. We quantified the effects of these three factors that relate land use to lake total phosphorus (TP) and total nitrogen (TN) in 346 north temperate lakes in 7 regions in Michigan, USA. We used a linear mixed modeling framework to examine the importance of spatial extent, lake hydrologic class, and region on models with individual lake nutrients as the response variable, and individual land use types as the predictor variables. Our modeling approach was chosen to avoid problems of multi-collinearity among predictor variables and a lack of independence of lakes within regions, both of which are common problems in broad-scale analyses of freshwaters. We found that all three factors influence land use-lake nutrient relationships. The strongest evidence was for the effect of lake hydrologic connectivity, followed by region, and finally, the spatial extent of land use measurements. Incorporating these three factors into relatively simple models of land use effects on lake nutrients should help to improve predictions and understanding of land use-lake nutrient interactions at broad scales.

## Introduction

Catchment land use has long been recognized as a major driver of nutrient concentrations in surface waters. For the past several decades, extensive field and process-based modeling research of individual catchments has improved understanding of the source and transport of nutrients from land to water. Transport of catchment-derived nutrients such as nitrogen (N) or phosphorus (P) to downstream water bodies is complex and depends on fine-scaled processes involving precipitation, runoff pathways, sediment delivery, flow-path length, soils, and general hydrogeologic setting [1–4]. Although transport rates can be estimated for catchments using process-based modeling, the data for model calibration are lacking for most waterbodies, especially when applying the models at broad spatial scales [5]. Therefore, to understand how catchment land use drives freshwater nutrients for the hundreds of thousands of water bodies for which we have only limited data and that are spread across regions and continents, we need models that work using widely available data, such as the percent of a catchment that is agricultural or urban land use, and that incorporate the most important factors for capturing variation in land use–freshwater nutrient relationships. In this paper, we study the effects of three of these factors that may minimize some of the variation around land use-freshwater nutrient relationships, and that may capture some of the fine-scaled transport processes described above–the spatial extent for measuring catchment land use; water body type; and regional differences in the *amount* of freshwater nutrients and the *effects* of land use on them.

First, because nutrient transport is influenced by the spatial configuration of land use types within a catchment, the spatial extent for measuring % land use around a water body is likely to be important. This effect has been demonstrated in a spatially-explicit modelling study of individual lakes that identified land use practices nearer to the lake shore or to connected streams as more influential in determining lake nutrient concentrations [6]. Spatial configuration can be defined to some degree by the ‘spatial extent’ at which land use is measured, which considers both the relative distance from the lake shoreline and the extent boundary used to delineate the area of land contributing nutrients to downstream water bodies [7]. Spatial extent is usually bounded using one of two methods. The first way is to use the topographical boundary (i.e., the catchment) of a water body, based on the assumption that all land uses within a topographical boundary contribute equally to material transport downstream. A second way is analogous to a riparian buffer, in which a spatial extent is bounded by an equidistant zone directly adjacent to a water body or to any connected upstream features and restricted to the topographical boundary (i.e., create equidistant zones of land around a lake within a catchment). Although the catchment scale is assumed to best represent land-water relationships, there is evidence that land use nearest a water body contributes disproportionately more nutrients due to shorter flow-paths that limit terrestrial nutrient uptake processes [8,9]. Because few studies have compared relationships between nutrients and land use quantified across a range of spatial scales, the optimal spatial extent for capturing nutrient transport to lakes is unclear.

Second, the relationships between land use and lake nutrients can differ by lake hydrologic connectivity, which has often been quantified by assigning lakes into hydrologic lake classes. We define a lake’s hydrologic class by the configuration of its surface water connections with upstream rivers and lakes [10,11]. Despite limnology’s rich history of lake classification, there have been relatively few studies that have tested whether relationships between nutrients and catchment land use differ by hydrologic class [12]. Relationships between lake nutrients and high-intensity catchment agriculture [13] and lake organic carbon and wetland cover [14,15] have both been found to differ among lakes with different connection strength to rivers. The presence of upstream lakes can also influence nutrient transport and the resultant concentrations of P or N in downstream lakes via a variety of mechanisms depending on the nutrient [11,16–18]. These results suggest that configuration of connected rivers and lakes provide additional explanatory power accounting for differences in relationships between land use and lake nutrients.

Finally, there is growing evidence that both lake nutrient concentration and relationships between land use and lake nutrients differ regionally depending on hydrogeomorphic setting, which can be defined by features such as climate, topography, and hydrogeology [19]. Regional differences have been well-documented for the concentrations of nutrients in streams, reservoirs, and lakes [5,20–24] and the magnitude of the effect of land use on freshwater nutrients [25–29].

**Fig 1:**
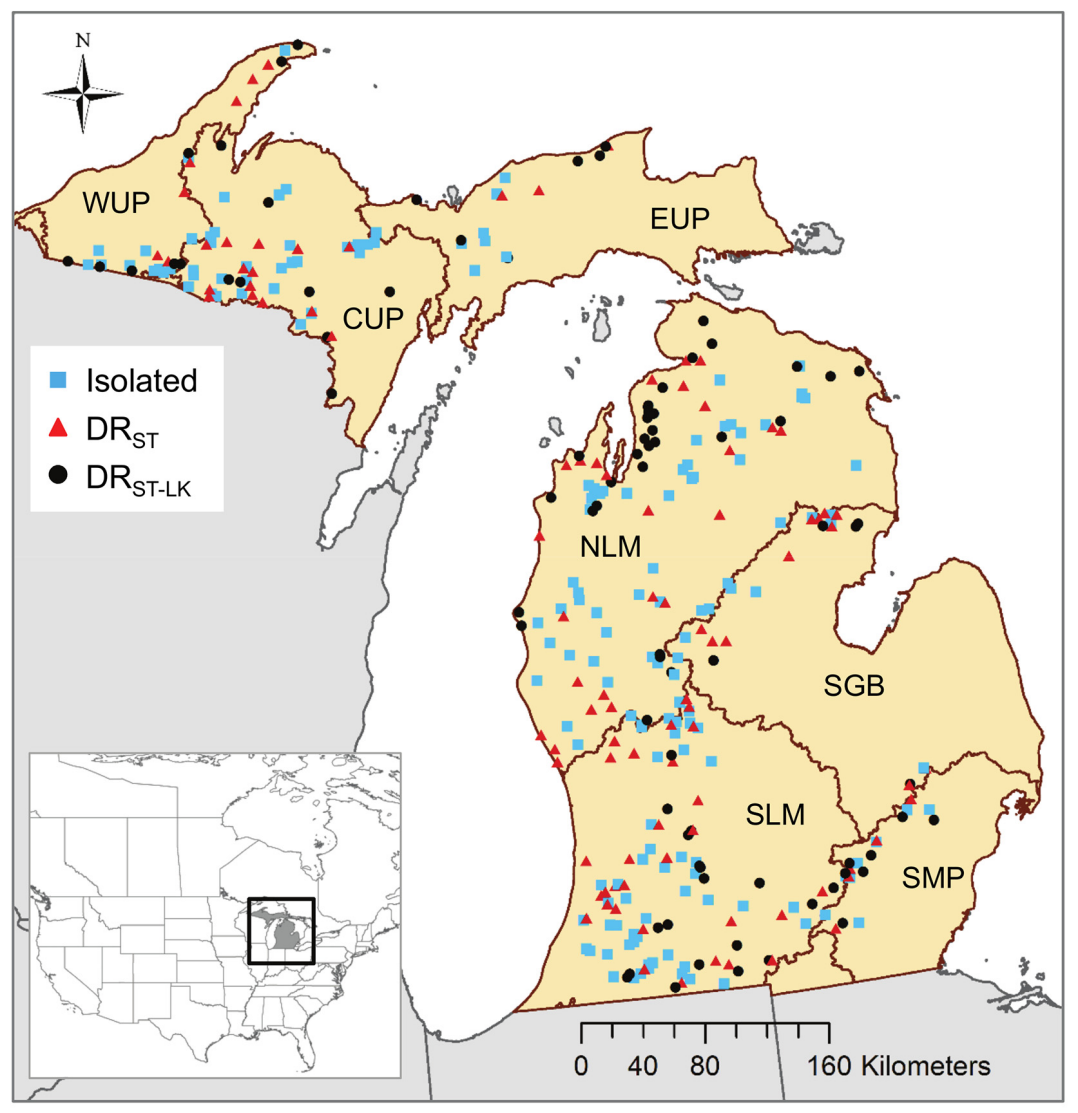
The location of the study lakes by lake class. The study area is Michigan USA and the map shows the regions used in all analyses (Ecological Drainage Units) delineated by brown lines within state boundaries delineated by grey lines. The 346 lakes are shown by lake hydrologic class (DR_ST_ are drainage lakes with stream inflows; DR_ST-LK_ are drainage lakes with stream inflows and at least one upstream lake ≥ 10 ha, see Fig 2 for details). The regions are labeled with a three-digit code as in Table 1. The inset shows the location of Michigan within North America.

In this paper, we use a linear mixed modeling framework to examine the importance of spatial extent, lake hydrologic class and region on relationships between land use (agriculture and urban) or land cover (forest and wetland) in a catchment and lake nutrients. Through our modeling approach, we address three specific questions: (1) At which spatial extents are land use/land cover (LULC) types related to lake nutrients? (2) Do lake hydrologic classes differ in their relationship between LULC and lake nutrients and in their optimal spatial extent for quantifying LULC?, and (3) Are there regional differences in lake hydrologic class-specific LULC effects on lake nutrients? In answering these questions, we explore LULC relationships for two nutrients, N and P, which are controlled by different dominant drivers and processes (e.g., denitrification; Bruesewitz et al. [30]) and, consequently differ in their relationships with LULC [31]. We compiled a database of 346 north temperate lakes ≥ 20 ha and ≥ 3 m maximum depth, in 7 regions in Michigan, USA (Fig 1) with lake nutrient and GIS-based LULC and hydrography data. For each lake, we assigned 1 of 3 hydrologic lake classes, delineated the catchment, and calculated LULC within the catchment for 6–8 spatial extents (depending on lake class).

## Methods

### Data

#### Nutrient data

Each one of 346 lakes was sampled once at some point between 1975–1982 during the summer stratified season (July–September) by the Michigan Department of Environmental Quality. These data match the temporal range of our LULC data (1978–1985). Standard methods for sample collection and laboratory processing were used. The lakes are all public lakes (with a public boat launch), are ≥ 20 ha and ≥ 3m in maximum depth. The entire lake and geographic database is available in the Dryad Repository, http://dx.doi.org/doi:10.5061/dryad.58445. We expect that variation in the year and date of sampling for different lakes combined with temporal variation in lake nutrients is likely to add error around our relationships. We have no way to quantify this error, but we have no evidence that the error would be systematic. Therefore, this source of error would not be expected to bias the spatial relationships that we are quantifying in this study.

#### Geographic data

We used Ecological Drainage Units [32] as our regional delineation, which has been shown to account for regional variation in lake nutrients in Michigan [20] and other US north temperate lakes [25]. Although our 346 lakes were not evenly distributed among the 7 regions (Table 1), our modeling approach (see below) can accommodate both unbalanced sample sizes across regions and low sample sizes within regions. We obtained LULC data from the Michigan Resource Information Service (from the Michigan Department of Natural Resources, Michigan Resource Inventory Program). These data contain detailed land cover classes based on interpretation of aerial photos taken between 1978 and 1985 at a resolution of 0.025 km^2^. We aggregated the data into the following four LULC types: agriculture, urban, forest, and wetland. To assign each lake to a hydrologic lake class (see below), we obtained stream hydrography and lake polygon data from the USGS National Hydrography Dataset at 1:24,000 resolution (http://nhd.usgs.gov/data.html).

**Table 1:** Numbers of study lakes by hydrologic lake class within each region.

Region^a^ (EDU)	Code	Isolated	DR_ST_	DR_ST-LK_	Total
SE Michigan interlobate and lake plain	SMP	8	5	6	19
Southeast Lake Michigan	SLM	48	32	20	100
Saginaw Bay	SGB	8	10	5	23
Northern Lake Michigan	NLM	52	26	32	110
Eastern upper peninsula	EUP	7	3	6	16
Central upper peninsula	CUP	28	16	8	51
Western upper peninsula	WUP	13	6	8	27
Total		164	97	85	346

DR_ST_ are stream-connected drainage lakes, DR_ST-LK_ are stream-lake connected drainage lakes. The location of the regions are shown in Fig 1 by code.

^a^ The regions used in this study are Ecological Drainage Units [32]. The three-digit codes are ordered roughly from southeast to northwest.

#### Lake hydrologic classification

**Fig 2:**
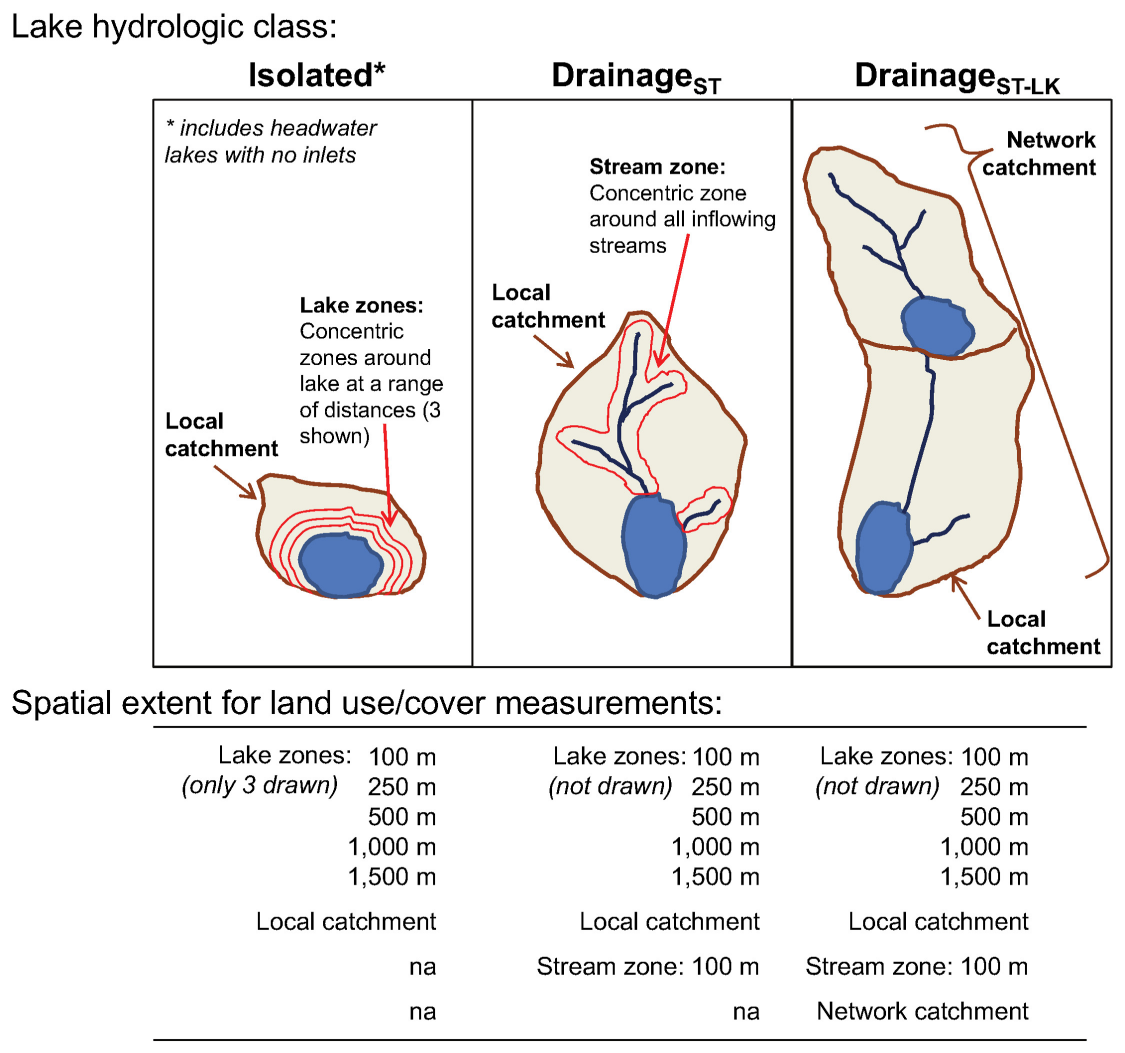
A description of lake hydrologic classes and the spatial extents for land use measurements. A diagram explaining the features of the 3 different lakes classes and the spatial extents tested in this study. The lake zones are shown on the isolated lake diagram, the stream zone is shown in the DR_ST_ diagram, and the network catchment is shown in the DR_ST-LK_ diagram. However, such scales are also quantified for some (but not all) of the different lake classes as depicted in the bottom half of the diagram. na is not applicable.

We developed a lake hydrologic classification (Fig 2) based on the presence of upstream surface hydrologic connections to each lake, and the presence of stream-connected upstream lakes (defined as a water body ≥ 10 ha). Our classification is based in part on two assumptions: (1) that upstream surface water connections influence nutrient transport to a lake, and (2) that 10 ha represents a size of stream-connected upstream lake that is sufficiently large to have an effect on downstream nutrient export. Unfortunately, there was little research with which to decide what size of lake influences downstream nutrient transport; consequently, we chose a size of lake that we judged as large enough to trap and retain water and nutrients, and for which it was logistically reasonable to develop watershed delineation methods. It is beyond the scope of this study to test alternative threshold lake sizes, but the question represents an area for future research. We classified the 346 lakes into one of three classes based on these upstream connections: isolated lakes with no stream inflows (n = 164; which included 53 lakes that were headwater lakes with no stream inflows but that contained an outflow); drainage lakes with stream inflows (DR_ST_; n = 97; which included both lakes with and without an outflow); and drainage lakes with stream inflows and at least one upstream lake ≥ 10 ha (DR_ST-LK_; n = 85; including both lakes with and without an outflow). Lake class influenced how spatial extents were defined because the presence and type of freshwater connections determines the type of spatial extents that can be quantified (see below for details).

#### Lake catchment delineations and LULC spatial extents

To delineate lake catchments and characterize each lake’s LULC at multiple spatial extents, we used the 30 m resolution USGS National Elevation Dataset (NED; http://ned.usgs.gov/). Existing watershed boundaries for rivers were used in the initial catchment delineation step and to constrain the catchment delineation process using the 1:24,000 Watershed Boundary Dataset (WBD; http://www.nrcs.usda.gov/wps/portal/nrcs/detail/national/technical/?cid=nrcs143_021630; USDA Natural Resources Conservation Service).

For all lakes, we defined the catchment as land draining directly to the lake, and land draining to inflow streams for DR_ST_ and DR_ST-LK_ lakes (Fig 2). For drainage lakes that had inflowing streams, but no upstream lakes (DR_ST_), the catchment included all upstream stream catchments. For drainage lakes that had inflowing streams and upstream lakes connected through stream connections (DR_ST-LK_), the catchment only included the stream catchments up until the next upstream lake (defined as a water body ≥ 10 ha). For DR_ST-LK_ lakes, we also calculated a network catchment, which we defined as the combined catchments of all connected upstream streams and lakes. For this class of lakes, we calculated LULC within the network catchment to represent an additional spatial extent.

Based on each lake’s catchment, we calculated equidistant zones around the lake that were constrained to be within the catchment boundaries (Fig 2). We examined a range of extents from the lake shoreline (100, 250, 500, 1000, and 1500 m). These zones are nested within each other such that the area of land within smaller zones is included in the measurement for the larger zones. Therefore, because we expected LULC in lake zones to be fairly well correlated with the other zones, we did not include more than one spatial extent in a model at a time. For all drainage lakes (DR_ST_ and DR_ST-LK_), we also calculated another spatial extent that we compared to the other zones, which was a single equidistant zone around the connected upstream streams within the lake’s local catchment. For this zone, we chose one extent width (100 m) based on past studies on streams.

### Statistical analyses

#### Hydrogeomorphic differences among lake hydrologic classes

To better understand how the lake classes differed from each other, we quantified a variety of hydrogeomorphic features that could influence LULC-nutrient relationships, including lake depth, lake area, catchment size, and length of inflow streams. We used a one-way ANOVA to compare differences among classes in these hydrogeomorphic factors.

#### Modeling overview

We used mixed-effects models to quantify the regional variation in the relationships between individual LULC types and either lake total phosphorus (TP) or total nitrogen (TN) by lake hydrologic class. All analyses were conducted in R [33]. For each nutrient, we computed 84 univariate models, with each nutrient as the response variable, and each of the four LULC types, at each of the 6–8 spatial extents, as the predictor variable. We conducted separate models for each of the 3 lake classes (i.e., for isolated lakes: 4 LULC types X 6 spatial extents = 24 models; for DR_ST_ lakes: 4 LULC types X 7 spatial extents = 28 models; for DR_ST-LK_ lakes: 4 LULC types X 8 spatial extents = 32 models). Because each of the lakes is nested within 1 of 7 geographic regions, we used mixed models, in which the lakes within regions could vary in mean nutrient concentration (i.e., a random intercept) and/or the lakes within regions could vary in the response of lake nutrients to differences in LULC (i.e., a random slope).

We conducted our analysis in two steps (described in greater detail below). First, we quantified the amount of lake nutrient variation occurring among regions. If there was a significant (p < 0.05) amount of among region variation, we allowed each region to vary in its mean lake nutrient concentration (i.e., a random intercept; see below). Second, we evaluated the potential for the effects of LULC on lake nutrients to vary regionally. If there was significant among-region variation in slopes a random slope was also included (see below). By creating and comparing univariate models, we avoided any problems of multicollinearity in the data (which are likely across spatial extents and LULC types). Although we performed multiple univariate analyses, we did not correct for family-wise type-I error rates because *a priori*, we were interested in univariate relationships and because we also examined the magnitude of the estimated effects and associated uncertainty to help identify ecologically meaningful relationships (i.e., we did not rely on p values alone for making inferences). Together, our 2 sets of 105 univariate mixed models fully explored the influence of lake hydrologic class, spatial extent, and region on lake nutrient–LULC relationships.

#### Non-varying LULC effects with varying intercepts by region

We first fit 6 unconditional (i.e., models with only a varying intercept term and no covariates) models for each lake class and nutrient combination (2 nutrients and 3 lake classes) to evaluate if there were among-region differences in average nutrient concentrations. We used these models to determine if it was necessary to account for regional differences in the intercepts of subsequent models. We calculated the intraclass correlation coefficient (ICC), which is the percent of the total variation among lakes that is among-region variation as opposed to within-region variation. Next, we fit the 168 models with a LULC covariate as a fixed effect (non-varying LULC slope parameter), and varying intercepts (assuming the unconditional model showed significant variation in average nutrient concentrations among regions). This model form allowed for regional differences in the average nutrient concentrations, but assumes the same relationship (slope) between the covariate and lake nutrient for all regions.

To compare results across the different spatial extents and lake classes for each nutrient-LULC combination, we calculated and plotted the R^2^, the fixed (non-varying slope) effect, and the 95% confidence interval (CI) of the fixed effect for each model. We deemed a relationship to be not ecologically relevant if the CI of the slope overlapped zero. We compared the patterns of these three measures among the 6–8 spatial extents for each lake class and LULC type. For example, if the slope of the LULC-nutrient relationship was similar across lake classes for each particular spatial extent, then we concluded that lake class does not matter when assessing the influence of spatial extent of that LULC type on the lake nutrient. We synthesized the results, separately for each lake class, in table form, highlighting the spatial extents for which there was evidence of ecologically relevant LULC-nutrient relationships (assessed as the spatial extents with the highest R^2^ values (within 0.1 of each other) and the largest effect sizes with the smallest CI’s that do not overlap zero). Similar to our comparisons among spatial extents, we considered lake classes to differ in their LULC-nutrient relationships (for a given LULC type) if the effect sizes and their CI’s did not overlap, and if the R^2^ values were > 0.1 apart.

#### Varying LULC effects among regions

For the second set of models, we examined whether the effects (i.e., slopes) of the lake nutrient–LULC relationships varied among regions within each of the 168 models, by fitting each model with a varying intercept and a varying slope for region. We estimated parameters using restricted maximum likelihood and used a likelihood ratio test to evaluate if there was evidence for differences in regional effects [34].

## Results

### Hydrogeomorphic differences among lake hydrologic classes

**Fig 3:**
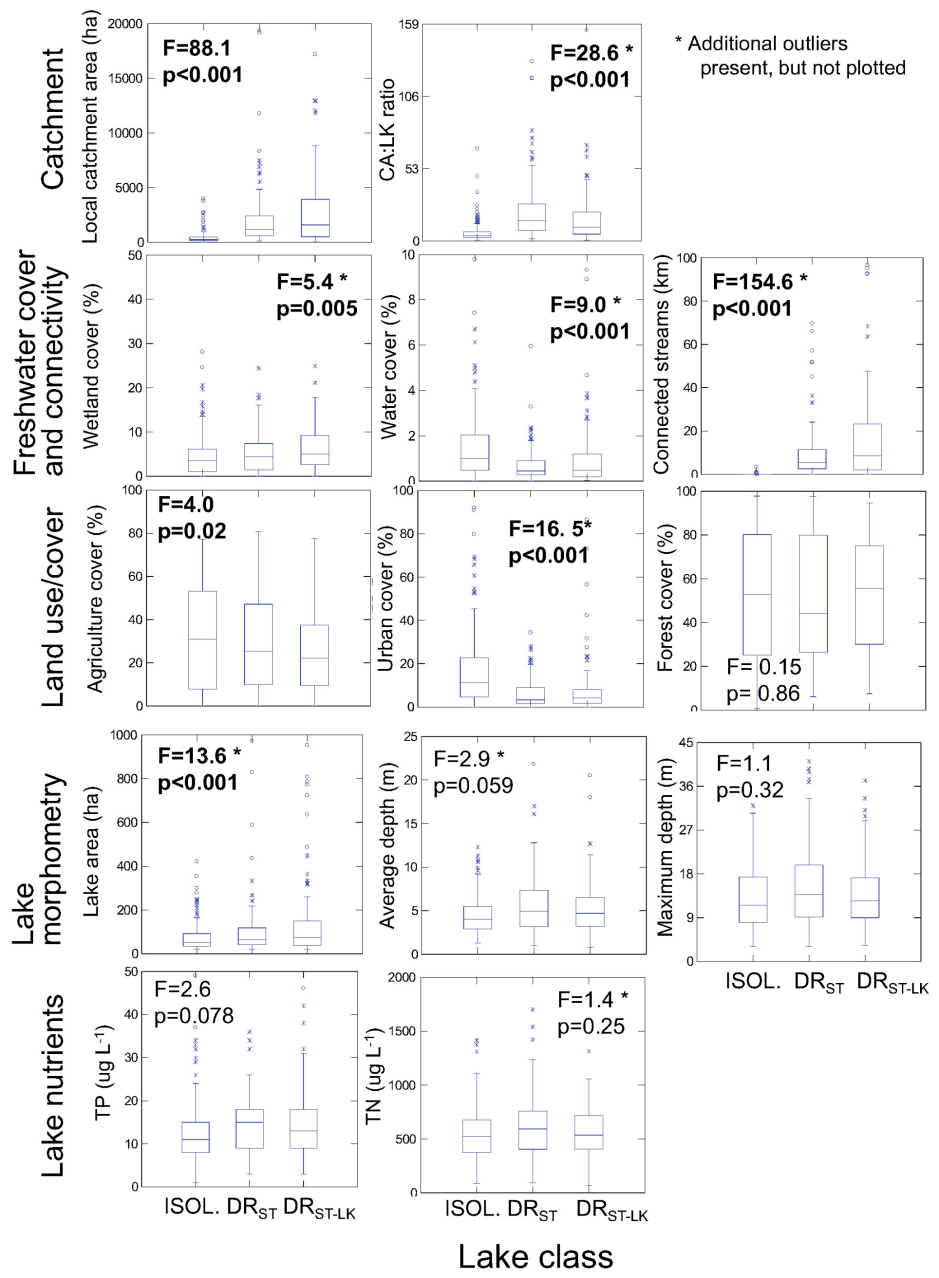
Box plots of lake and landscape characteristics by lake class. Where relevant, the landscape features are calculated for the local catchment spatial extent (see methods for details). The lake classes are ordered from left to right in order of increasing complexity of connections to other water bodies. Outliers are not plotted for all figures. The *F* statistic and *p* value are shown for a one-way ANOVA to test the difference among the three lake classes for each variable. Statistics in bold are significant at *p* ≤ 0.05. Samples sizes are 164 for isolated lakes, 97 for DR_ST_ lakes, and 85 for DR_ST-LK_ lakes.

The study lakes included both shallow and deep lakes, and ranged from oligotrophic to eutrophic, although the majority of lakes were in the oligotrophic to mesotrophic range (Fig 3). Our hydrologic lake classification captured several important hydrogeomorphic differences among lakes (Fig 3). As would be expected, the presence of upstream lakes and streams, upon which the classification is based, as well as the presence of wetlands and small water bodies (<10 ha), differed among classes. Additionally, catchment properties such as area differed among lake classes, as did the ratio of catchment area to lake area (CA:LK), which is strongly correlated with water residence time for lakes [35,36]. Isolated lakes generally were smaller than the drainage lakes, although lake depth did not differ greatly among lake classes. Importantly, lake nutrients did not differ significantly among classes, indicating that lake hydrology alone is not sufficient to distinguish lakes by nutrient concentration. Finally, there was evidence that LULC differed across the lake classes, at least for human-dominated land use (agriculture and urban), although variation within classes was large.

### Non-varying LULC effects by lake hydrologic class and spatial extent

**Fig 4:**
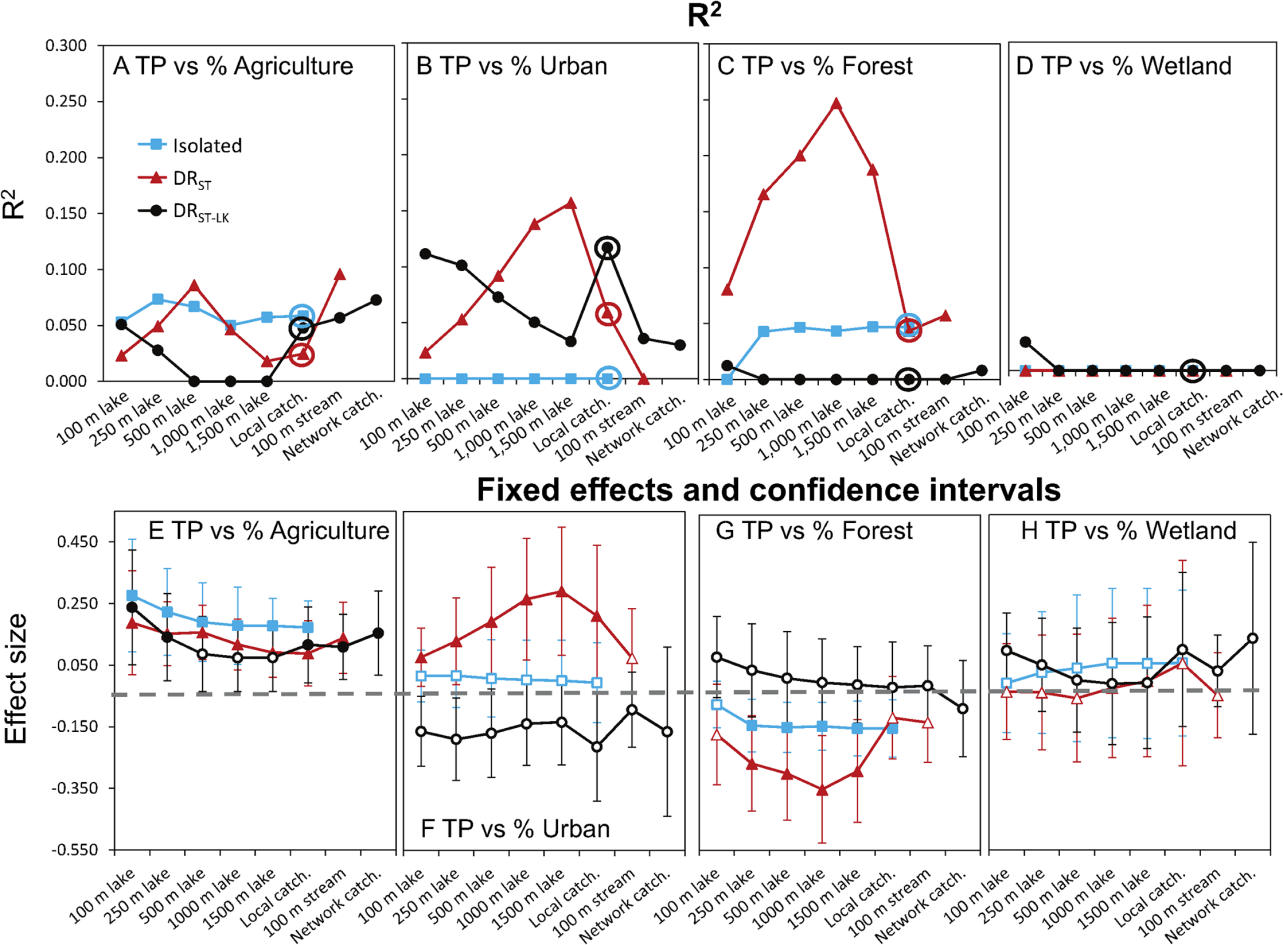
Model fit and effect sizes for the fixed effects in models of land use-lake total phosphorus (TP). For the relationships between TP and individual LULC types by lake class and spatial extent, the R^2^ (A-D) and slopes (E-H) from mixed-models with random intercepts and fixed effects of the LULC type. Individual models were fit for each lake class and spatial extent of a particular LULC type combination (i.e., for % agriculture land use, 21 individual models were fit). In plots E-H, the error bars are confidence intervals (CI’s) of the slopes from the above models. Filled symbols are for the fixed effect slopes in which the CI does not overlap zero. Note, we show lines connecting the points of each lake class for ease of comparison across lake classes only.

**Fig 5:**
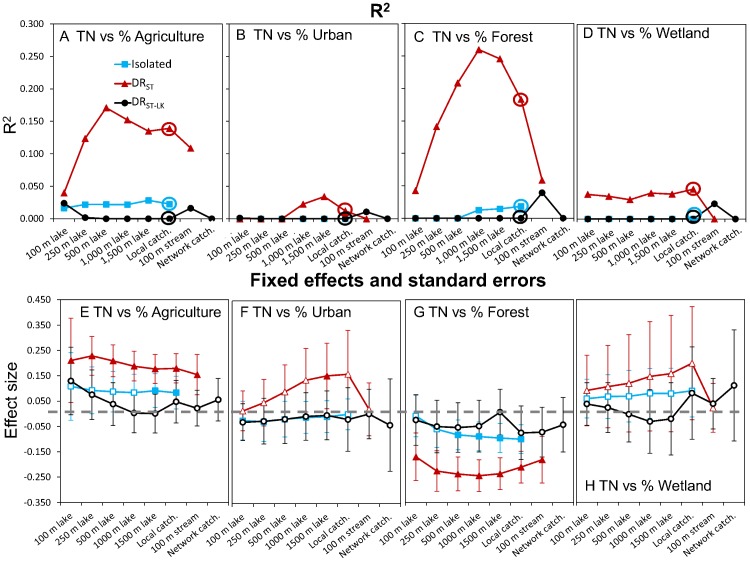
Model fit and effect sizes for the fixed effects in models of land use-lake total nitrogen (TN). For the relationships between TN and individual LULC types by lake class and spatial extent, the R^2^ (A-D) and slopes (E-H) from mixed-models with random intercepts and fixed effects of the LULC type. Individual models were fit for each lake class and spatial extent of a particular LULC type combination (i.e., for % agriculture land use, 21 individual models were fitted). In plots E-H, the error bars are confidence intervals (CI’s) of the slopes from the above models. Filled symbols are for the fixed effect slopes in which the CI does not overlap zero. Note, we show lines connecting the points of each lake class for ease of comparison across lake classes only.

**Fig 6:**
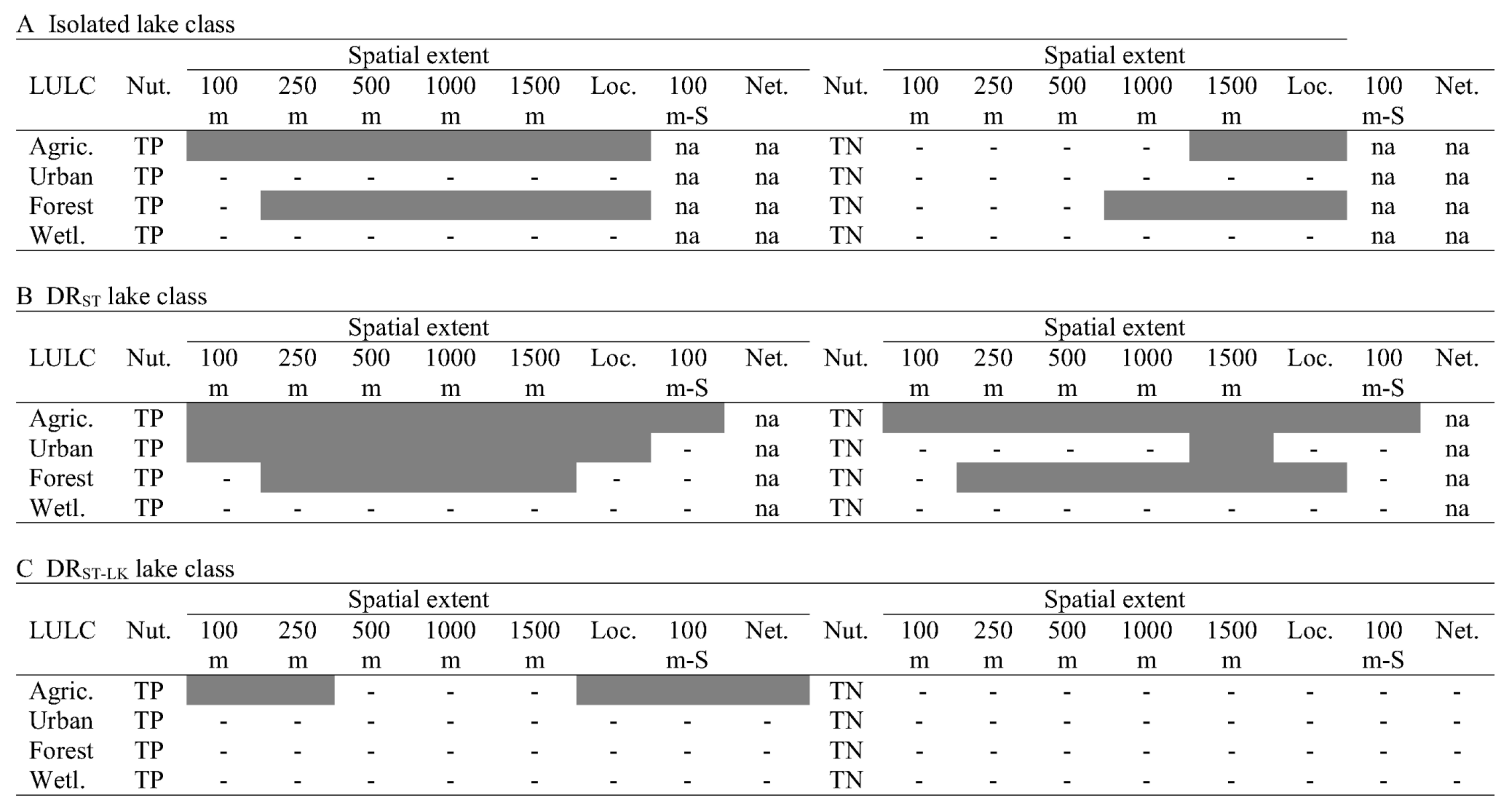
Synthesis of results by spatial extent and nutrient. The dark grey shading corresponds to evidence for a stronger relationship at one or more spatial extents than at the other non-shaded spatial extents based on both R^2^ and the absolute value of the slope (larger positive or negative number indicating a stronger effect). Dashes indicate a lack of an ecologically relevant relationship (in which either the slope CIs overlap zero or an R^2^ value close to zero). LULC is the land use/land cover type, Wetl. is wetland, Nut. is nutrient, Loc. is local catchment, 100m-S is the 100 m zone around inflowing streams, Net. is the network catchment. The DR_ST_ lake class includes drainage lakes with streams flowing in; and the DR_ST-LK_ lake class includes drainage lakes with streams flowing in and upstream lakes ≥ 10 ha.

We found variation in the average concentration of nutrients among regions; the percent variation in nutrients at the regional scale (ICC) varied among the hydrologic lake classes from 2–16% for TP and from 18–31% for TN (Table 2). These results supported the inclusion of a varying region intercept in subsequent mixed models. We also found evidence that LULC-nutrient relationships differed among lake classes and spatial extents, and between the two nutrients (Figs 4–6). Among the 168 models, R^2^ values ranged ~0 to ~0.25; fixed effect (slope) values also differed in magnitude and direction among models (Figs 4 and 5). Overall, we found stronger LULC-nutrient relationships for TP than for TN (Fig 6). For both nutrients, the estimated effects of urban land use and wetland cover had large uncertainty for all 3 lake classes, which limited our ability to evaluate the effect of spatial extent of measuring these LULC types on their relationships with nutrients. In contrast, we found the strongest relationships between lake nutrients and forest land cover and agricultural land use, which had opposing effects on lake nutrients and which are correlated with each other, both of which we expected. The correlations between these two LULC types at the catchment scale were -0.75, -0.87, and -0.74 for isolated, DR_ST_, and DR_ST-LK_ lakes respectively (S1 Table).

**Table 2:** Results from the unconditional mixed-effect models.

		ICC (%)^a^	
Lake hydrologic class	*n*	TP	TN
Isolated	164	12	34
DR_ST_	97	2	18
DR_ST-LK_	85	16	31

The models are for total phosphorus (TP) and total nitrogen (TN) with varying intercepts and no covariates, testing for regional differences in average lake nutrient concentration. Lake classes as for Table 1.

^a^ ICC = intraclass correlation coefficient, the percent of the total variation among lakes that is among-region variation.

Comparing spatial extents, we found little evidence that measuring LULC at the local catchment spatial extent was better (i.e., resulted in a stronger relationship with nutrients) than measuring LULC at other spatial extents. Rather, we found LULC measured at this spatial extent was correlated to LULC measured at other spatial extents (S1 Table). In fact, for agricultural land use, measurements for all spatial extent pairings were correlated with each other (p < 0.001). However, some spatial extents were more correlated than others. For example, agricultural land use measured in the 100 m lake zone was the least correlated to agricultural land use measured at other spatial extents, particularly the local catchment scale (r = 0.64, 0.60, and 0.53 for isolated, DR_ST_, and DR_ST-LK_ lakes, respectively). Correlations in LULC among spatial extents of measurement resulted in similarities among spatial extents in the relationships between LULC and lake nutrients. For example, for the relationships in which catchment LULC was found to be related to either nutrient, there were 1–5 other spatial extents that were equally related to the nutrient (Fig 6). Interestingly, there were several cases in which we detected no relationship between a catchment LULC type and either TP or TN: for isolated lakes, urban and wetland cover for both nutrients; for DR_ST_ lakes, forest and wetland cover for TP, and urban and wetland cover for TN; and for DR_ST-LK_ lakes, all land uses and covers except for agriculture for TP (Fig 6).

Comparing lake classes, we found the most ecologically relevant LULC-nutrient relationships for DR_ST_ lakes, and the fewest for DR_ST-LK_ lakes (Fig 6). In particular, the strongest LULC-nutrient relationships, measured as both R^2^ and slope magnitude, occurred in DR_ST_ lakes for forest land cover versus TP and TN (Figs 4 and 5). LULC-nutrient relationships were particularly weak for DR_ST-LK_ lakes for forest land cover and wetland cover vs TP and all LULC types vs TN (Figs 4 and 5).

Comparing effect sizes, we found that there were differences among lake classes in some cases, but not all. For example, for the TP-urban land use relationships, the effect was positive for DR_ST_ lakes and negative for DR_ST-LK_ lakes, although in the latter case, the CIs for the slopes overlapped zero (Fig 4). In addition, for the TN-agriculture land use and TN-forest land cover relationships, DR_ST_ lakes had ecologically relevant differences in slopes compared to both other lake classes; isolated lakes had intermediate slopes, and DR_ST-LK_ lakes had no relationship (Fig 5).

To varying degrees, we found that both lake class and spatial extent of LULC measurement was important. For example, the relationships between LULC and lake TP were similar (in both R^2^ and effect size) across many of the spatial extents of LULC measurement, for both agricultural land use and forest land cover. This similarity among spatial extents of LULC measurement was strongest for isolated and DR_ST_ lakes, and is especially striking for the 100m lake zone and lake catchment spatial extents because it is at these spatial extents that agricultural land use measurements are least correlated with each other (S1 Table), suggesting that different mechanisms underlie these relationships. For DR_ST-LK_ lakes, relationships between agricultural land use and lake TP varied substantially among spatial extents of LULC measurements. Agricultural land use was related to lake TP within the 100 and 250 m lake zones, the 100 m stream zone, and both catchment extents, but no ecologically relevant relationships between agricultural land use and lake TP occurred for LULC measured at the intermediate 1,000 and 1,500 m extents.

### Varying LULC effects by region

**Fig 7:**
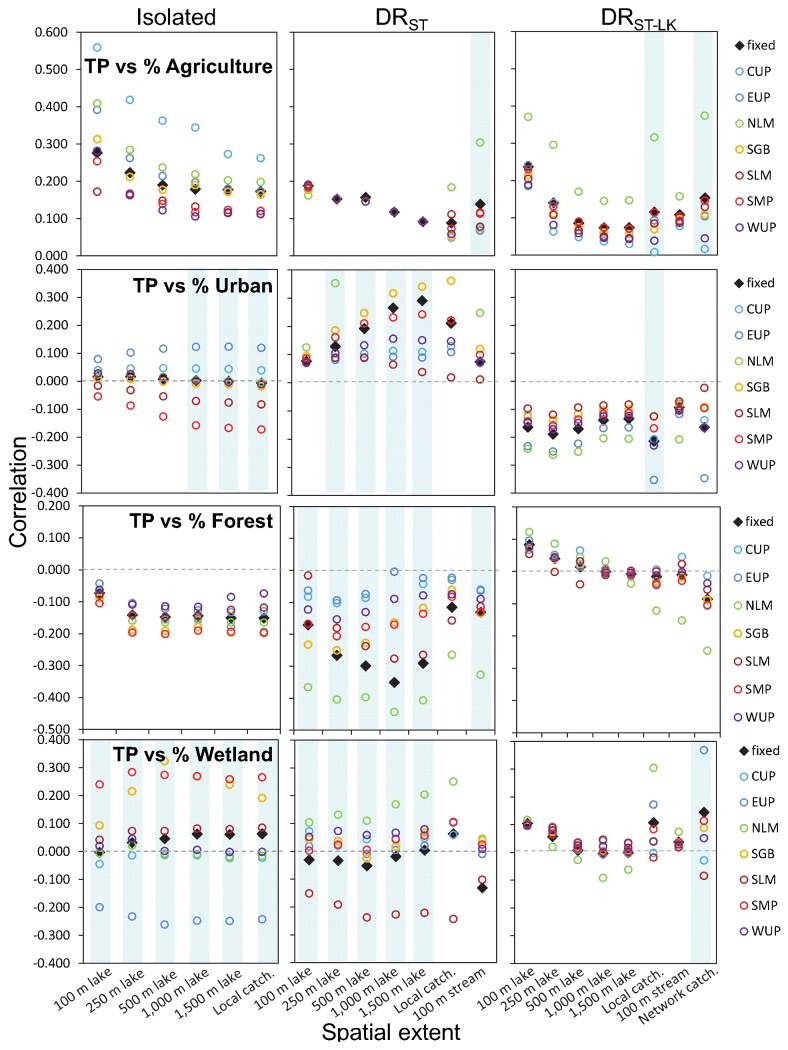
Region-specific slopes in models of land use-lake total phosphorus (TP). For the relationships between TP and individual LULC types by lake class, spatial extent, and region, the region-specific slopes (colored circles, without the CI’s, for clarity) from mixed-effects models with varying intercepts and varying slopes of the LULC type. The light blue vertical bars indicate whether the varying slope parameter in this model was significant using a likelihood ratio test. We also show the fixed effect slopes including the non-significant estimates from Figs 4 and 5 as black diamonds for comparison only. The region codes are as in Table 1.

**Fig 8:**
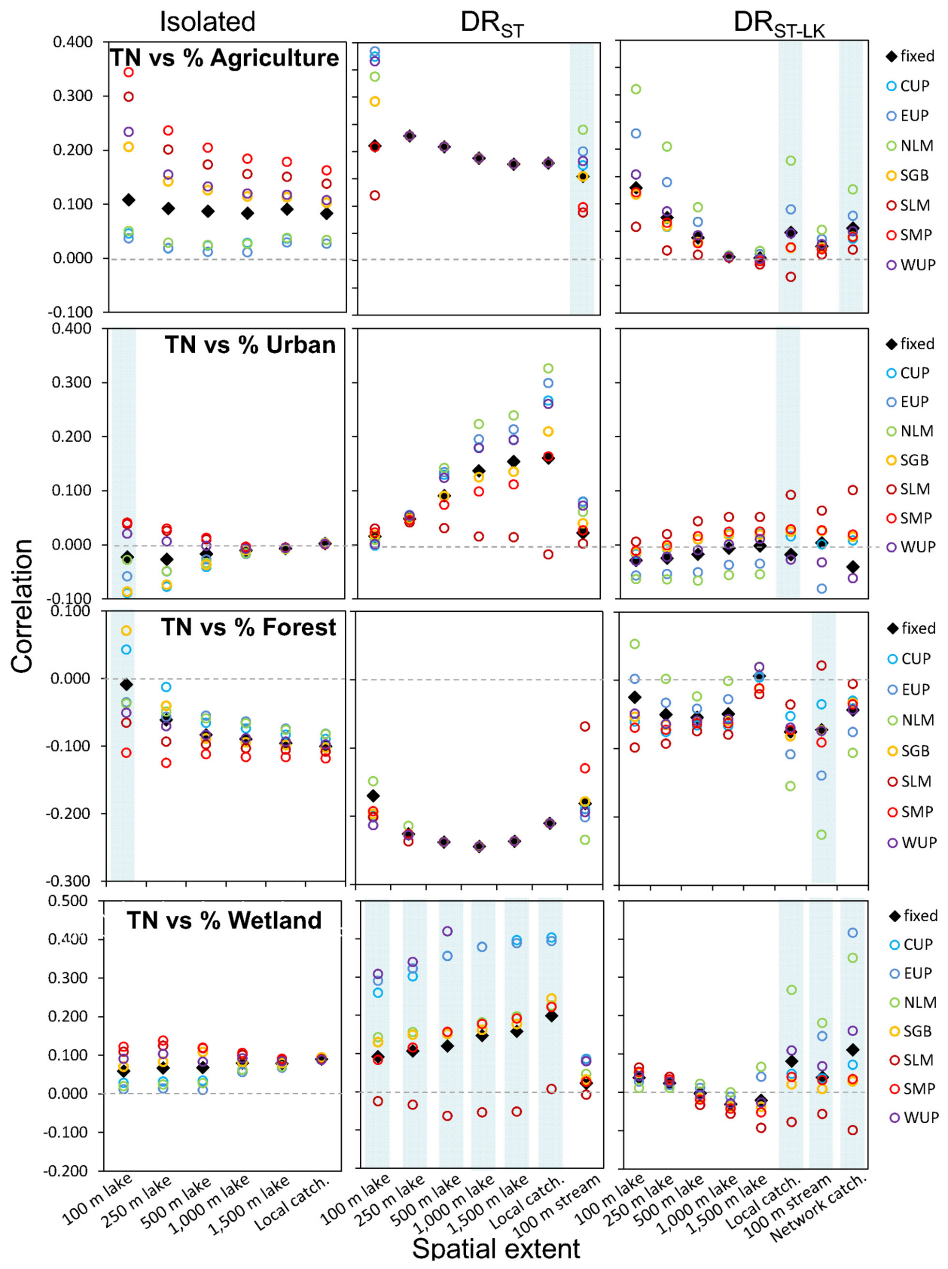
Region-specific slopes in models of land use-lake total nitrogen (TN). For the relationships between TN and individual LULC types by lake class, spatial extent, and region, the region-specific slopes (colored circles, without the CI’s, for clarity) from mixed-effects models with varying intercepts and varying slopes of the LULC type. The light blue vertical bars indicate whether the varying slope parameter in this model was significant using a likelihood ratio test. We also show the fixed effect slopes including the non-significant estimates from Figs 4 and 5 as black diamonds for comparison only. The region codes are as in Table 1.

We found regional variation in the effects of LULC on lake nutrients, but only for some LULC-nutrient-lake class combinations and for some spatial extents (Figs 7 and 8). For both nutrients, there were regional differences for urban land use and forest and wetland cover effects for most of the lake classes and spatial extents. Additionally, for TN, there were regional differences for agricultural land use for most contexts.

When there was significant regional variation in effects for one or more of the spatial extents, the sign of the relationship did not usually differ among regions (e.g., all slope estimates were positive or negative). However, for the two cases in which region-specific slopes differed in sign, we also found small or non-existent fixed effects (Figs 4 and 5). The first case corresponds to the relationship between TP and urban land use in isolated lakes; three of the spatial extents had significant region-specific slopes, yet there were no significant fixed effects measured at any extent. Two regions had positive slopes, two regions had negative slopes, and a range of regions had slopes close to zero. Similarly, for relationships between TP and TN and wetland cover, all but one relationship between either nutrient and wetlands across the three lake classes (isolated lakes for TN) had at least one significant region-effect for slopes at any spatial scale. As in the example described above, some regions had negative wetland-nutrient slopes, some had positive, and some had slopes that were small or zero (Figs 7 and 8). These strong region-specific effects in opposite direction cancel each other out when lakes in all regions are analyzed together (as in the fixed-effects models) and can help to explain many of the observed fixed effects that were close to zero for wetland relationships with either nutrient and for most lake hydrologic classes.

### Synthesis

**Fig 9:**
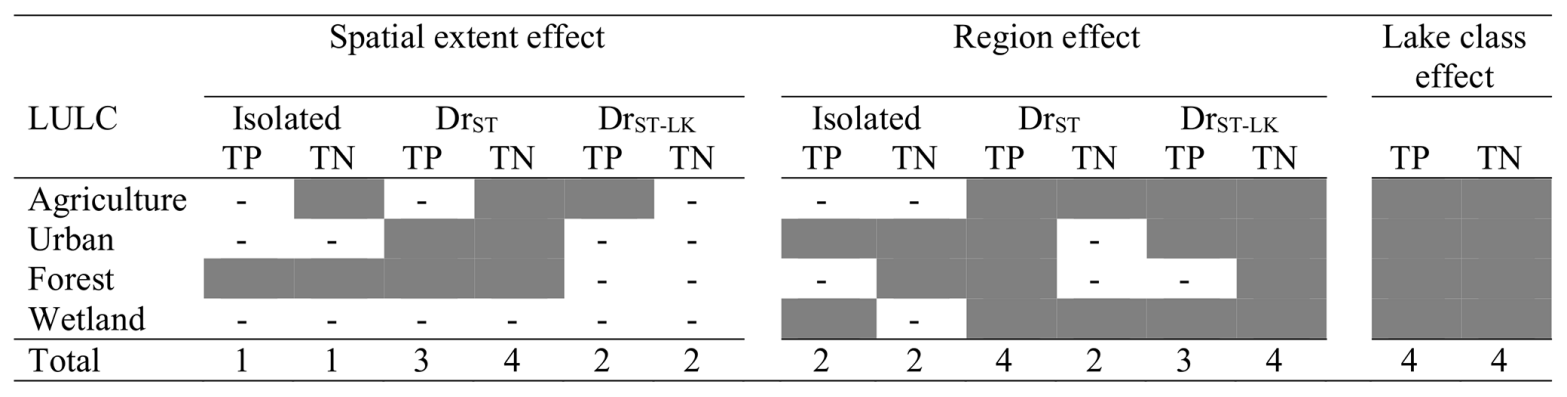
Synthesis of results for the relationship between lake nutrients and LULC. Categories and descriptions as for Fig 6. The dark grey shading corresponds to either spatial extent (the fixed effects), region (the random effects), or lake class (comparison across models) being important for a specific land use/cover (LULC)-nutrient combination based on visual inspection (and some statistical evaluation) of graphs and model output. The numbers at the bottom row indicate the number of the tested LULC types (4 total) in each column that were shown to be important predictors of lake nutrient concentrations. The dashes indicate no relationship.

Overall, we found that there were ecologically important effects of spatial extent, region, and lake hydrologic class for many of the LULC-lake nutrient combinations (Fig 9), although there were differences in the strength of evidence for the effects of these factors. For example, we found the most consistent effects of lake hydrologic class on LULC-lake nutrient models. In contrast, we found the least strong evidence for a spatial extent effect for measuring LULC effects on lake nutrients. In other words, models for one spatial extent did not seem to greatly outperform models for other spatial extents based on standard errors of the effect sizes (i.e., there was much overlap across the different spatial extent measurements of LULC). Finally, we found moderately-strong evidence for a region effect on LULC-lake nutrient models in many cases.

## Discussion

We found that all three factors, spatial extent, lake hydrologic class, and region, are likely to influence the relationships between LULC and lake nutrients. Specifically, our analyses demonstrate that: (1) The strength of the LULC- nutrient relationships differed by LULC spatial extent; (2) The LULC-nutrient effect sizes and percent variation explained differed by lake hydrologic class in most cases; and (3) There were regional differences in some of the LULC-nutrient relationships indicating that effects of particular LULC types were not consistent across regions. In many cases, our findings were consistent with predictions based on mechanistic understanding generated at local scales, but we can point to patterns that emerge at broad spatial extents of analysis that cannot be fully accounted for by local process models or their broad scale surrogates. Because our results demonstrate that LULC-nutrient relationships differed among spatial extent-hydrologic lake class-region combinations, all three factors should be incorporated into broad-scale LULC models predicting lake nutrients.

Although the R^2^ values from our models were low, they do not include any other predictor variables that are known to influence lake nutrients, and only include a single LULC type. In addition, the highest values that we report are within the middle range of R^2^ values reported from regressions using a global dataset by region [5]; and are close to other studies modeling lake or reservoir nutrients from LULC [13,29,35,37]; but are much lower than those of Jones et al. [38] who examined relationships for reservoirs. Many of the above studies are based on multiple samples taken throughout the summer (and sometimes from multiple years) that are averaged; however, our models are built on single-samples. Therefore, the generally lower R^2^ values in our study may be due to high temporal variation that we could not account for. Nevertheless, for many of our models, the 95% CI’s of the slope values are relatively small, and do not overlap zero, suggesting the relationships are ecologically relevant.

### Relationships between LULC and lake nutrients at different spatial extents

Some of our results comparing the strengths and effect sizes of relationships along a spatial-extent gradient were unexpected. Past work suggests that LULC measured at the lake catchment spatial extent would capture consistently more variation in lake nutrients relative to other spatial extents of measurement, as found by Nielsen et al. [37] for lake nutrients, and by Hunsaker and Levine [9] for stream nutrients. In addition, much past work has emphasized the importance of the catchment scale for lake or reservoir nutrients [5,23,38–40]. Only a few studies have compared alternate spatial extents for studying the relationship between LULC and freshwater N and P across broad scales. Nielsen et al. [37] measured LULC-lake nutrient relationships across a range of zone extents including 25, 50, 100, 200, and 400 m, and the entire catchment in a set of Danish lakes. They concluded the catchment scale was most appropriate; however, they did not test larger zone sizes as we did here, and in fact we found larger zones (1,000 and 1,500 m) to be very well related to lake nutrients. Jones et al. [38] found that riparian LULC did not explain any additional variation in reservoir nutrients beyond that variation due to catchment LULC. In contrast, Fraterrigo and Downing [41] found that nutrients in reservoirs with low transport capacity (and generally smaller watersheds) were more strongly related to riparian LULC than catchment LULC; and nutrients in reservoirs with high transport capacity (and larger watersheds) were more strongly related to catchment LULC than riparian LULC. In sum, it is likely that there is no one optimal scale to measure LULC across different water body classes and regions for the purpose of predicting freshwater nutrient concentrations. Further multi-scaled studies and tests are needed to better resolve this issue.

In our study lakes, the catchment scale had moderately strong relationships with lake nutrients, but so did the other spatial extents, especially the 1,000 and 1,500 m lake zones. Because for most of the LULC types, the different spatial extents are correlated with each other, particularly the 1,000 and 1,500 m zones with catchment LULC (S1 Table), we cannot determine the underlying mechanisms operating at the different spatial extents [7,38,42]. Further work is needed to identify and understand possible mechanisms that may be operating at different spatial extents. Nevertheless, our results support the assertion of Devito et al. [43] who argue that the focus on the catchment scale for studying hydrologic responses (which are a major control of nutrient transport from land) is not always warranted. Our results show that a range of different spatial extents may capture the dominant hydrologic processes that ultimately influence nutrient transport from land to water and that the best spatial extent to measure LULC can differ by lake class, and in fact, it also may differ by region. These results make sense in light of the fact that regions differ in hydrogeomorphic settings, climate, hotspots of biogeochemical activity, and thus, connections between land and water. Consequently, it may be unrealistic to presume that a single model and spatial extent best applies across broad geographic study regions.

### The role of hydrologic connectivity in LULC-lake nutrient relationships

Our analyses provide evidence that lake hydrologic classes differ in their relationships to the dominant spatial extent and regional control of lake nutrients. Other studies have found similar lake classifications to be effective for examining the relationship between freshwater nutrients or DOC and LULC. Gergel et al. [14] examined the relationship between wetland cover and DOC for lakes divided into two hydrologic classes (isolated and drainage) based on connections to streams and found the two lake classes to have large differences in the strength of the relationships; in fact, the differences between lake classes were larger than the differences among the different tested spatial extents used to measure wetland cover. Also, Abell et al. [13] found that the effect size of intensive pasture use on nutrients in the catchment was stronger in lowland lakes compared to headwater lakes in New Zealand. Finally, Read et al. [44] compared the same three lake hydrologic classes that we used in this analysis when building more complete models that related local and regional drivers of lake nutrients across the continental US; they found that not only did model fits differ among lake classes, but the significant predictor variables differed across the lake classes as well.

Comparing across the lake hydrologic classes, we were not surprised that we detected the fewest number of ecologically important relationships in the lake class that has upstream lakes (DR_ST-LK_). A recent modeling study quantifying the effect of upstream lakes on LULC-TP relationships showed that as the area of upstream lakes increases, the model fits get increasingly worse [11]. Likewise, in simulations by Epstein et al. [17], the effect of upstream lakes in a chain on nutrients downstream depended on whether nutrients were being predicted in the first downstream lake in a chain of connected lakes, or farther down the chain. Although all upstream lakes reduced nutrients in downstream lakes, only the first lake served as a sink for P and a source of N, whereas additional lakes downstream were sources of both N and P. If this latter result is a general property of lake networks, then our relatively simple designation of the presence of any amount of upstream lake may result in highly variable relationships between lake nutrients and LULC among DR_ST-LK_ lakes, depending on variation in the number and size of upstream lakes. Therefore, it is likely that our lake classification needs revision to effectively capture these spatial relationships related to complex sources and sinks of nutrients in lake networks. This result is important because this lake hydrologic class (DR_ST-LK_) is well-represented in glaciated North America and likely elsewhere. For example, for all lakes in Michigan ≥ 20 ha (n = 2010), 30% have upstream lakes (Soranno, unpubl. data), and these larger lakes are the ones most often sampled by management agencies [45]. A better understanding of the underlying controls that connected and upstream freshwaters have on nutrients originating from land is needed to better model the effects of LULC in larger catchments that often have complex freshwater networks. Our results, coupled with past research, support the contention that lake hydrologic classes are functionally different in their response to external drivers and provide a potential broadly useful factor to consider when quantifying LULC effects on lake nutrients.

Importantly, our simple lake hydrological classification does not capture differences in nutrient concentrations among the 346 lakes in our analysis. Rather, our hydrologic lake classes capture many differences among lakes that influence nutrient loading such as the length of inflowing streams, the area of upstream lakes, wetland cover, catchment area, and the ratio of catchment to lake area ratio (CA:LK; Fig 3), many of which are strongly related to water residence time (WRT). This classification appears to create functionally useful classes that are related to variables that are particularly important for measuring LULC effects on lakes, specifically WRT and catchment area. Water residence time has long been considered essential for understanding controls on lake nutrients [13,40,46,47]. Although we did not directly measure WRT, it has a strong negative correlation to CA:LK. Based on CA:LK, inferred WRT was shortest for DR_ST_ lakes and longest for isolated lakes, with DR_ST-LK_ lakes intermediate. And, in our analyses the strongest relationships and the largest effect sizes occurred between agricultural land use and lake nutrients in lakes likely to have short WRT’s and agriculture land use, similar to findings elsewhere [13].

### Regional differences in LULC effects on lake nutrients

As the spatial extent of studies of freshwaters increases to include broad-scale research questions and global pressures such as climate change, it becomes necessary to account for regional, and in some cases, continental, differences in fundamental ecological relationships [25,27]. A hierarchical approach similar to what we used here not only accounts for regional differences, which is necessary statistically [48], but also identifies regional divergence in fundamental relationships such as LULC effects on freshwater nutrients. We found that regional differences in LULC effects on lake nutrients obscured relationships and interfered with our ability to quantify fixed effects of LULC cover types on lake N or P. In several instances, LULC in different regions had opposite (or undetectable) effects on lake nutrients; and, when data from all regions were combined to estimate a fixed effect, the result was an effect that was not ecologically (or statistically) important. This result was common enough (i.e., TP versus urban land use for isolated lakes; and TP and TN versus wetland cover for most lake classes) to warrant further consideration in future studies. A more common result consisted of no detectable effects in some or many regions, but strong effects detected in a few regions. Although low sample sizes in some regions may be responsible in part for lack of a detected LULC effect on lake nutrients, as indicated by the large CIs around many of the slopes, it also may be the case that some drivers are less relevant in some regions than in others. Given the tendency for mostly significant results to be published [49], our approach can help to identify not only the regions where nutrients may be strongly controlled by LULC, but also the regions where nutrients may be more strongly controlled by drivers other than LULC.

Other studies have documented regional differences in the effects of LULC on freshwaters. For example, Fergus et al. [27] found regional differences in the relationships between wetland cover and both lake TP and water color; Cross and Jacobson [29] found regional differences in relationships between LULC and lake TP; and Johnes et al. [50] concluded that region-specific LULC export coefficients were required to best model river nutrients in England and Wales. An important consideration in comparing these studies is the spatial extent of the entire study area because as the number and heterogeneity of regions increases, then it is more likely that the regional scale will be important. For example, Xenopoulos et al. [15], who studied relationships from different continents, found that models developed for one region did not apply to other regions and continents. Identifying the broad-scale factors that determine when models from some regions can effectively be applied to other regions remains an important challenge for ecologists and managers.

## Conclusions

We have shown how three factors can contribute to variation around relationships between LULC and lake nutrients. First, the spatial extent within which the LULC is measured matters; and although LULC measured at the entire catchment scale is effective for many LULC-lake nutrient relationships, it is not effective for all relationships, and other spatial extents are either equally or more effective at capturing variation in lake nutrients for some relationships. Second, some lake hydrologic classes have poor to no relationship between LULC and lake nutrients, particularly isolated lakes and lakes with upstream lakes. Finally, regional differences in LULC-lake nutrient relationships can be large, and sometimes have opposite effects (i.e., some positive and some negative). Incorporating these three factors into relatively simple models of LULC effects on lake nutrients should help to improve predictions and understanding of LULC-lake nutrient interactions at broad scales.

## Supporting Information

S1 Table Correlation matrix of agricultural land use measured at the different spatial extents by lake class. (DOCX)
