# Supplementary material for: Effects of Land Use on Lake Nutrients: The Importance of Scale, Hydrologic Connectivity, and Region
Source: PLoS One. 2015 Aug 12;10(8):e0135454. doi: 10.1371/journal.pone.0135454 (PMC4534397; doi:10.1371/journal.pone.0135454)
Supplement: S1 Table — (DOCX) [file pone.0135454.s001.docx]

S1 Table. **Correlation matrix of agricultural land use measured at the different spatial extents by lake class.**

A Isolated lake hydrologic class

|  | 100 m | 250 m | 500 m | 1,000 m | 1,500 m | Local |
| --- | --- | --- | --- | --- | --- | --- |
| 100 m | 1 |  |  |  |  |  |
| 250 m | 0.839 | 1 |  |  |  |  |
| 500 m | 0.753 | 0.971 | 1 |  |  |  |
| 1,000 m | 0.702 | 0.927 | 0.979 | 1 |  |  |
| 1,500 m | 0.682 | 0.911 | 0.965 | 0.994 | 1 |  |
| Local | 0.645 | 0.886 | 0.941 | 0.973 | 0.987 | 1 |

B DR_ST_ lake hydrologic class

|  | 100 m | 250 m | 500 m | 1,000 m | 1,500 m | Local | 100 m-ST |
| --- | --- | --- | --- | --- | --- | --- | --- |
| 100 m | 1 |  |  |  |  |  |  |
| 250 m | 0.882 | 1 |  |  |  |  |  |
| 500 m | 0.756 | 0.959 | 1 |  |  |  |  |
| 1,000 m | 0.680 | 0.894 | 0.959 | 1 |  |  |  |
| 1,500 m | 0.644 | 0.857 | 0.925 | 0.984 | 1 |  |  |
| Local | 0.600 | 0.818 | 0.887 | 0.942 | 0.965 | 1 |  |
| 100 m-ST | 0.604 | 0.751 | 0.783 | 0.809 | 0.799 | 0.812 | 1 |

C DR_ST-LK_ lake hydrologic class

|  | 100 m | 250 m | 500 m | 1,000 m | 1,500 m | Local | 100 m-ST | Net-work |
| --- | --- | --- | --- | --- | --- | --- | --- | --- |
| 100 m | 1 |  |  |  |  |  |  |  |
| 250 m | 0.907 | 1 |  |  |  |  |  |  |
| 500 m | 0.778 | 0.953 | 1 |  |  |  |  |  |
| 1,000 m | 0.702 | 0.891 | 0.970 | 1 |  |  |  |  |
| 1,500 m | 0.643 | 0.838 | 0.930 | 0.981 | 1 |  |  |  |
| Local | 0.533 | 0.732 | 0.811 | 0.856 | 0.896 | 1 |  |  |
| 100 m-ST | 0.399 | 0.501 | 0.524 | 0.587 | 0.636 | 0.761 | 1 |  |
| Network | 0.556 | 0.729 | 0.784 | 0.806 | 0.832 | 0.925 | 0.682 | 1 |

(A) Isolated lake hydrologic class; (B) DR_ST_ lake hydrologic class, which are stream-connected drainage lakes; (C) DR_ST-LK_ lake hydrologic class, which are are stream-lake connected drainage lakes. All correlations are significant at p < 0.001. The spatial extents are as for Figs. 4-5.
